# Research methodology and characteristics of journal articles with original data, preprint articles and registered clinical trial protocols about COVID-19

**DOI:** 10.1186/s12874-020-01047-2

**Published:** 2020-06-22

**Authors:** Mahir Fidahic, Danijela Nujic, Renata Runjic, Marta Civljak, Filipa Markotic, Zvjezdana Lovric Makaric, Livia Puljak

**Affiliations:** 1 Faculty of Medicine, University of Tuzla, Tuzla, Bosnia and Herzegovina; 2 Department of Public Health, Faculty of Medicine, Osijek, Croatia; 3 Department of Public Health, Humanities and Social Sciences in Biomedicine, Faculty of Dental Medicine and Health, Osijek, Croatia; 4 University of Split School of Medicine, Split, Croatia; 5 Center for Evidence-Based Medicine and Health Care, Catholic University of Croatia, Ilica 242, 10000 Zagreb, Croatia; 6 Croatian Agency for Medicinal Products and Medical Devices, Zagreb, Croatia; 7 Department of Epidemiology, Croatian National Institute of Public Health, Zagreb, Croatia

**Keywords:** SARS-CoV-2, COVID-19, Coronavirus, Original research, Clinical trial, Protocol, Preprint

## Abstract

**Background:**

The research community reacted rapidly to the emergence of COVID-19. We aimed to assess characteristics of journal articles, preprint articles, and registered trial protocols about COVID-19 and its causal agent SARS-CoV-2.

**Methods:**

We analyzed characteristics of journal articles with original data indexed by March 19, 2020, in World Health Organization (WHO) COVID-19 collection, articles published on preprint servers medRxiv and bioRxiv by April 3, 2010. Additionally, we assessed characteristics of clinical trials indexed in the WHO International Clinical Trials Registry Platform (WHO ICTRP) by April 7, 2020.

**Results:**

Among the first 2118 articles on COVID-19 published in scholarly journals, 533 (25%) contained original data. The majority was published by authors from China (75%) and funded by Chinese sponsors (75%); a quarter was published in the Chinese language. Among 312 articles that self-reported study design, the most frequent were retrospective studies (*N* = 88; 28%) and case reports (*N* = 86; 28%), analyzing patients’ characteristics (38%). Median Journal Impact Factor of journals where articles were published was 5.099.

Among 1088 analyzed preprint articles, the majority came from authors affiliated in China (51%) and were funded by sources in China (46%). Less than half reported study design; the majority were modeling studies (62%), and analyzed transmission/risk/prevalence (43%).

Of the 927 analyzed registered trials, the majority were interventional (58%). Half were already recruiting participants. The location for the conduct of the trial in the majority was China (*N* = 522; 63%). The median number of planned participants was 140 (range: 1 to 15,000,000). Registered intervention trials used highly heterogeneous primary outcomes and tested highly heterogeneous interventions; the most frequently studied interventions were hydroxychloroquine (*N* = 39; 7.2%) and chloroquine (*N* = 16; 3%).

**Conclusions:**

Early articles on COVID-19 were predominantly retrospective case reports and modeling studies. The diversity of outcomes used in intervention trial protocols indicates the urgent need for defining a core outcome set for COVID-19 research. Chinese scholars had a head start in reporting about the new disease, but publishing articles in Chinese may limit their global reach. Mapping publications with original data can help finding gaps that will help us respond better to the new public health emergency.

## Background

On December 31, 2019, the World Health Organization (WHO) China Country Office was informed by the Chinese authorities of a series of pneumonia cases with unknown etiology (unknown cause) in Wuhan, Hubei, China, with clinical presentations that greatly resembled viral pneumonia. The Chinese authorities have isolated a causal agent on 7 January 2020, which was identified as a new type of coronavirus (novel coronavirus, nCoV) [1], titled “severe acute respiratory syndrome coronavirus 2” (SARS-CoV-2) and the disease it causes “coronavirus disease” (COVID-19) [2].

After emerging in China, the virus has spread rapidly throughout the world. On April 29, 2020, there were 3,162,438 confirmed cases throughout the world, with 219,287 deaths due to COVID-19 [3]; these numbers were escalating rapidly day by day.

The research community has responded rapidly to this new threat to humanity. On March 19, 2020, a simple search of PubMed, using the most common terms associated with the new virus and disease (coronavirus OR COVID-19 OR COVID 19 OR SARS-CoV-2), revealed that almost 2000 such articles were published since December 1, 2019. However, cursory browsing of those articles indicated that the majority of them appeared to be editorials, news, and opinions.

This is the third coronavirus epidemic in the third millennium, after severe acute respiratory syndrome (SARS) in 2002 and Middle East respiratory syndrome (MERS) in 2012; it is highly pathogenic and requires urgent action in the research community [4]. Mapping research methodology of published original studies and registered clinical trials since the outbreak of pandemic will help researchers in getting a better overview of relevant studies published thus far and how fast the research community has responded to the new health threat immediately following the outbreak.

This study aimed to identify and classify published original research studies, preprint articles and registered clinical trials regarding the SARS-CoV-2 and COVID-19 from December 1, 2019, until March/April 2020, the period which would correspond to the first months following the outbreak. We did not include an earlier period because the first official report about the new disease was submitted to the WHO on December 31, 2019 [1].

## Methods

### Protocol and registration

We defined protocol for this review prospectively and, for transparency, the protocol was published on Open Science Framework (OSF), URL: https://osf.io/dzvxc/ after the final draft of the protocol was endorsed by all co-authors, and before the commencement of any work.

### Eligibility criteria

We included original studies of any study design that reported original data related to the virus SARS-CoV-2 and disease it causes, COVID-19, from December 1, 2019, onwards. We searched for records without language restrictions. We excluded articles reporting editorials, news, opinions, and other types of articles that did not report original research data. All excluded articles were tabulated, with references, and reasons for exclusion. We included articles posted on preprint servers medRxiv and bioRxiv, as well as registered protocols of clinical trials about SARS-CoV-2 and COVID-19.

### Information sources

To retrieve published original studies, we used publicly available WHO Database of publications on coronavirus disease (COVID-19) [5]. The WHO has created this Database based on searches of bibliographic databases and hand-searching of tables of contents of relevant journals, as well as other scientific articles that came to their attention [5]. We conducted a separate initial search of MEDLINE using common keywords related to COVID-19 (coronavirus OR COVID-19 OR COVID 19 OR SARS-CoV-2), and we found a similar number of records as presented in the WHO database. We downloaded the full database in Excel and EndNote format on March 19, 2020.

We downloaded a list of preprint articles published in medRxiv and bioRxiv on April 3, 2020. The download was made via web site of the medRxiv (https://www.medrxiv.org/), where there is a link to „COVID-19 SARS-CoV-2 preprints from medRxiv and bioRxiv“. We accessed registered protocols of clinical trials from the WHO International Clinical Trials Registry Platform (WHO ICTRP) on April 7, 2020. For both preprint articles and clinical trial registrations we did not conduct any searches, as these information sources had pre-curated collections devoted to COVID-19, and they do not publish other types of content. Two authors screened preprint articles and clinical trial registrations to make sure they were about COVID-19.

### Selection of sources of evidence

For published articles, two review authors screened all records (titles/abstracts) retrieved from the WHO Database. For each record, they noted their opinion on whether the study was eligible or not, and if not what was the reason (not related to the topic, not an original study report). We retrieved full texts of eligible or potentially eligible studies and two review authors independently screened them. For each full text, reviewers recorded their opinion about study eligibility, and reasons for exclusion (not related to the topic, not an original study report). Disagreements between reviewers in the second screening phase, evaluating full texts, were resolved via discussion or involvement of other authors. For preprint articles and registered clinical trials, one author verified their eligibility because they were downloaded from curated collections dedicated to COVID-19.

### Data charting process

For published studies, one review author extracted the data and another author verified data extraction. Disagreements were resolved via discussion, or involvement of the third author if necessary. We extracted the following data, related to characteristics of articles and journals, in a standardized format for each eligible study: date of publication, journal, Journal Impact Factor (JIF) for the year 2018, country of the authors’ affiliation (whole count method was used, whereas each country was counted once, regardless of the number of authors from an individual country), unit of analysis (humans, animal models, etc.) study aim, number of authors, self-reported study design, a thematic group in line with categories used by The Evidence for Policy and Practice Information and Co-ordinating Centre (EPPI-Centre) [6], information about study funding, study sponsor name, study sponsor country. We classified all studies into three groups based on study design: observational, experimental, and evidence synthesis. For studies in languages other than English, we used Google Translate, as it has been shown that it is a viable, accurate tool for data extraction from non-English articles used in evidence syntheses [7]. For any uncertainties, we planned to contact native speakers of languages other than English. This was necessary only regarding an article in Persian.

For preprint articles, we extracted the following data: title, DOI, link to online article, abstract, number of authors, country of affiliation (using the whole country method), self-reported study design, a thematic group in line with categories used by EPPI-Centre [6], information about study funding, study sponsor name, study sponsor country.

For registered protocols, we analyzed the following data: clinical trial registry where the protocol was primarily registered, recruitment status, minimal and maximal age of participants, sex of eligible participants, self-reported study type, a location where the study will be conducted, and primary outcome.

### Synthesis of results

We analyzed data using descriptive statistics, frequencies, and percentages.

## Results

### Articles with original data published in scholarly journals

Among the first 2118 articles on COVID-19 published in scholarly journals, 533 (25%) contained original data. We have excluded 1585 articles for the following reasons: not original research (*N* = 1386), duplicate articles (*N* = 118), unrelated to the topic (*N* = 56), correction (*N* = 18), preprint server publication (*N* = 4), study protocol (*N* = 2), and retraction (*N* = 1). The list of analyzed and the list of excluded studies is available on OSF (https://osf.io/dzvxc/). The first article was published on January 21, 2020. The majority of articles were published in English (*N* = 401; 75%); a quarter was published in Chinese (*N* = 131; 24%), and one article was published in Persian.

The median number of authors was 7 (range: 1 to 63). Articles were published in 207 different journals. The highest number of articles was published in the Journal of Virology (*N* = 33; 6.1%) (Table 1). For 377 articles published in journals with a JIF, the median JIF was 5.099 (range: 0.364 to 70.670).

**Table 1:** Characteristics of analyzed journal articles with original data

Variable (N of denominator)	N (%)^a^
Journals (*N* = 533)	
Journal of Medical Virology	33 (6.1)
Journal of Infection	18 (3.3)
International Journal of Infectious Diseases	16 (3.0)
Clinical Infectious Diseases	15 (2.8)
Radiology	14 (2.6)
Other	437 (82)
Country in the author affiliation (*N* = 533)	
China	402 (75)
USA	62 (12)
UK	21 (3.9)
Japan	20 (3.7)
Italy	19 (3.5)
Other	9 (1.7)
Self-reported study design (*N* = 312)	
Retrospective study	88 (28)
Case report	86 (28)
Case series	46 (15)
Modelling	18 (5.7)
Systematic review with or without meta-analysis	16 (5.1)
Other	58 (18.6)
Thematic classification (*N* = 456)	
Case reports – patients	173 (38)
Transmission/risk/prevalence	104 (23)
Genetics/biology	57 (13)
Health impacts	54 (12)
Diagnosis	41 (9)
Other	27 (5.9)
Country of study funders (*N* = 268)	
China	202 (75)
USA	13 (4.9)
Japan	11 (4.1)
Korea	5 (1.9)
Canada	4 (1.5)
Other	33 (12.3)

^a^Denominator is provided in the first column, as “N of trials with reported variable”; for some variables due to rounding the sums may not be exact 100%, for variables we presented only five most frequent categories

The median number of countries in the authors’ affiliations was 1 (range: 1 to 9). Authors from 48 countries authored the articles, the majority of affiliations were from China (*N* = 402; 75%), followed by the USA (*N* = 62; 12%) (Table 1).

In 312 (58%) journal articles, authors self-reported study design. The most common self-reported study designs were retrospective study (*N* = 88; 28%) and case report (*N* = 86; 28%) (Table 1). Our classification of articles in three major groups showed that there were 503 (94%) observational studies, 19 (4%) evidence syntheses of various types, and 11 (2%) experimental studies.

Among the 533 articles, 456 were in the EPPI-Centre living map of evidence; the majority were classified as case reports (*N* = 173; 38%) (Table 1). In 381 (71%) articles unit of analyses were humans; in the majority (*N* = 236; 62%) only adults were included. Declaration about study funding was reported in 324 (60%) of the journal articles; among those, there were 268 (83%) articles that reported that the study received funding. Sponsors were most commonly from China (*N* = 202; 75%) (Table 1).

### Preprint articles

From the exported 1102 preprint articles we excluded 4 that were withdrawn and 10 that were about SARS and MERS; we included the remaining 1088 preprint articles in the analysis. The list of analyzed preprint articles is available on OSF (https://osf.io/dzvxc/). The majority was posted on medRxiv (Table 2). The first preprint article on COVID-19 was posted on bioRxiv on January 19, 2020; it reported a mathematical model of transmission of the novel virus [8], the first article was posted on medRxiv on January 24, 2020; it reported early estimation of epidemiological parameters and epidemic predictions regarding the novel virus [9].

**Table 2:** Characteristics of analyzed preprint articles

Variable (N of denominator)	N (%)^a^
Preprint server (*N* = 1088)	
medRxiv	842 (77)
bioRxiv	246 (23)
Country in the author affiliation (*N* = 1088)	
China	563 (52)
USA	298 (27)
UK	92 (8.4)
Italy	51 (5.3)
Hong Kong	43 (3.9)
Other	41 (3.7)
Self-reported study design (*N* = 494)	
Modelling	306 (62)
Retrospective study	59 (12)
Cross-sectional study	35 (7.1)
Cohort study	22 (4.4)
Systematic review with or without meta-analysis	21 (4.3)
Other	51 (10)
Thematic classification (*N* = 1088)	
Transmission/risk/prevalence	470 (43)
Health impacts of COVID-19	163 (15)
Genetics/biology	127 (12)
Diagnosis	101 (9.2)
Treatment development	84 (7.7)
Other	137 (13)
Country of study funders (*N* = 681)	
China	312 (46)
USA	107 (16)
UK	14 (2)
Japan	13 (1.9)
China and USA	11 (1.6)
Other	224 (33)

^a^Denominator is provided in the first column, as “N of trials with reported variable”; for some variables due to rounding the sums may not be exact 100%, for variables we presented only five most frequent categories

The median number of authors was 7 (range: 1 to 178). The most common country in the authors’ affiliations was China (51%) (Table 2). In 494 (45%) preprint articles, authors self-reported study design. The most common self-reported study design was a modeling study (Table 2).

The most frequent thematic classification of the preprint articles was transmission/risk/prevalence (43%; Table 2). Study funding was reported in 681 (63%) of the preprint articles. The majority of funders were from China and the USA (Table 2).

### Registered clinical trials

By April 7, 2020, there were 927 clinical trials indexed on WHO ICTRP. The list of analyzed registered trials is available on OSF (https://osf.io/dzvxc/). The first trial was indexed on January 27, 2020. The majority (*N* = 581; 63%) of trials were primarily registered on the Chinese Clinical Trials Registry (ChiCTR), followed by ClinicalTrials.gov (*N* = 286; 30%). Few trials were primarily registered with other platforms (Table 3).

**Table 3:** Characteristics of analysed clinical trial registrations

Variable (N of trials with reported variable)	N (%)^a^
Clinical trial registry (*N* = 927)	
Chinese Clinical Trials Registry (ChiCTR)	581 (63)
ClinicalTrials.gov	286 (30)
EU Clinical Trials Register	21 (2.2)
Australian New Zealand Clinical Trials Registry (ANZCTR)	9 (1)
ISRCTN	8 (0.9)
IRCT	8 (0.9)
Other	14 (1.5)
Recruitment status (*N* = 915)	
Not recruiting	453 (50)
Recruiting	441 (48)
Authorized	21 (2.2)
Minimal age of participants (*N* = 744)	
18 years	532 (72)
0 years	26 (3.5)
14 years	18 (2.4)
16 years	15 (2)
1 year	13 (1.7)
Other	140 (15)
Maximal age of participants (*N* = 663)	
Not applicable/no upper limit	197 (30)
80 years	59 (9)
75 years	55 (8.2)
90 years	42 (6.3)
65 years	32 (4.8)
Other	278 (42)
Eligibility of participants based on sex (*N* = 921)	
Both men and women	892 (97)
Only men	18 (1.9)
Only women	11 (1.2)
Self-reported study type (*N* = 927)	
Interventional	535 (58)
Observational	303 (33)
Diagnostic test	35 (3.8)
Observational (patient registry)	19 (2)
Epidemiological research	10 (1)
Other	25 (2.7)
Location of trials located in single countries (*N* = 825)	
China	622 (63)
United States	33 (4.0)
France	21 (2.5)
Italy	17 (2.1)
United Kingdom	10 (1.2)
Other	122 (15)
Tested interventions (*N* = 535)	
Hydroxychloroquine	39 (7.2)
Chloroquine	16 (3.0)
Tocilizumab	10 (1.9)
Lopinavir/ritonavir combination	10 (1.9)
Convalescent plasma	9 (1.7)
Other	451 (84)

^a^Denominator is provided in the first column, as “N of trials with reported variable”; for some variables due to rounding the sums may not be exact 100%, for variables we presented only five most frequent categories

Recruitment status was available for 915 (99%) of registered protocols, and among them about half were either “not recruiting” or “recruiting” (Table 3). None of the trials retrieved from WHO ICTRP were labeled as “withdrawn” in the recruitment status. However, 38 (4%) of protocols were labeled as “Cancelled” in the name of the study; all these protocols were indexed primarily in ChiCTR.

In 744 trials, the minimal age of participants was specified. In the majority, the minimal age of participants was 18 years (*N* = 532; 72%) (Table 3). In 663 trials, information about the maximum age of participants was provided. In about a third of them (*N* = 197; 30%), it was specified that there was no upper age limit (Table 3). In 921 protocols there was information about the inclusion of participants based on sex; the majority (*N* = 892; 97%) reported they will include both men and women (Table 3).

The majority of registered trials were described as interventional (*N* = 535; 58%), followed by descriptor “observational” (*N* = 322; 35%) (Table 3). Among registered “trials”, there were even 7 that were described as “basic science” (Table 3).

The median number of planned study participants was 140 (range above zero: 1 to 15,000,000). For eight protocols, the planned number of participants in the WHO ICTRP data was zero; we checked web sites of all those protocols and found that five of them were from ClinicalTrials.gov where they were labeled as withdrawn, the remaining three were from ChiCTR, whereas one had information about the number of patients in the wrong field, but the remaining two did not have any explanation for zero number of patients.

Five protocols did not have any information about the number of participants; two were canceled protocols from ChiCTR, two were protocols labeled as “Expanded access status” in ClinicalTrials.gov, and we were unable to verify the fifth because the web link was not functional. In interventional studies, the median number of planned participants was 108 (range from 1 to 55,000), while in the observational median was 200 (range from 8 to 15,000,000). Three protocols reported that the planned number of participants was higher than one million.

In 825 registrations, the location, where the trial will be conducted, was reported. Only 20 (2.4%) reported that the trial will be conducted in more than one country. Most of the trials for which it was reported they will be conducted in a single location were located in China (*N* = 522; 63%), followed by the United States (*N* = 33; 4%) (Table 3).

In 535 trial protocols described as interventional, 532 (99%) provided information about the primary outcome. Most of the protocols (*N* = 260; 49%) had multiple primary outcomes that were not described as composite. In studies with a single or composite primary outcome (*N* = 272), highly heterogeneous primary outcomes were used (details about registered trials are available on OSF; https://osf.io/dzvxc/). Few outcomes were used more commonly. The most commonly used outcome was time to recovery, used in 40 (15%) protocols, and phrased differently such as “time to clinical recovery”, “time to clinical improvement”, “time to disease recovery”, “time to remission”, “clinical recovery time”, etc. The second most common outcome was mortality, found in 23 (8.4%) protocols with a single or composite primary outcome, described variously as mortality, all-cause mortality, in-hospital mortality, or mortality at certain time points (28 days, 30 days, 60 days).

In registered trials of interventions, various heterogeneous interventions were tested; the most frequently studied interventions were hydroxychloroquine (*N* = 39; 7.2%) and chloroquine (*N* = 16; 3%) (Table 3).

## Discussion

The research community has responded swiftly to COVID-19 in terms of scholarly dissemination output. The earliest date of onset of COVID-19 symptoms was reported as December 1, 2020 [10], and December 8, 2019 [11]. Our study shows that within about 3 months since the earliest reported date of onset of symptoms, more than two thousand articles were published in scholarly journals, a quarter of which had original data. Within 4 months from the public announcement [11] about the new disease, 1100 preprint articles were published and almost 1000 clinical trials registered.

The majority of studies came from China, which is understandable, as the disease originated there. Thus, Chinese scientists had a head start in exploring the disease. The majority of the first studies with original data, that were published in scholarly journals, had observational study design, which is understandable, as interventional studies usually take more time to be completed. However, the research community has responded rapidly with designing and registering clinical trials on COVID-19.

Even though the majority of journal articles with original data were published in English, a quarter was published in the Chinese language; this is concerning because those manuscripts may likely have valuable data, but they will be difficult to read and access by an audience that does not speak Chinese. Furthermore, this may prove challenging for conducting evidence syntheses; if the authors conducting systematic reviews and similar studies are unable to access or translate studies published in Chinese, those studies may not be included in evidence syntheses, thus contributing to biased evidence syntheses. Some authors of evidence syntheses deliberately upfront exclude articles published in languages other than English [12]; our results indicate that this may not be advisable in the evidence syntheses about COVID-19.

The median JIF of published articles was 5.099, which is rather high; it indicates that early articles were published in many high-impact journals, even if they described case reports, or case series, because of the novelty of the disease. It is likely that those journals were also able to accommodate submissions about COVID-19 quickly and organize rapid peer-review, and that those were journals with short turnaround times; journals with professional staff would be in a better position to adapt quickly to publishing novel topic of interest, compared to journals depending on volunteer staff.

While the majority of early articles about COVID-19 in scholarly journals were observational, mostly case reports, the predominant type of early articles about COVID-19 articles published on preprint servers included modeling studies. This might be early view of studies that will be soon published in peer-reviewed journals, but it remains to be seen how many of those preprint articles will actually pass the scrutiny of peer-review. It is possible that the massive production of modeling studies is leading to difficulties with publishing them, and that authors post those studies on a preprint server, to make their work publicly available. A large number of articles on preprint servers that we analyzed could be due to calls for authors to make their work publicly available in preprint servers along with submitting articles to peer-reviewed scholarly journals; there were even suggestions that submission to a preprint should be the default for all submissions [13].

The majority of registered trials we analyzed were registered in the Chinese registry of clinical trials, which is contrary to the report that ClinicalTrials.gov contains most of the global trial registrations [14], also, the overwhelming majority of registered trials we analyzed were conducted in China.

Although the aim of this study was not an in-depth analysis of outcomes and interventions that were used in registered trials about COVID-19, our analysis of those trials indicates both the novelty of the disease as well as methodological shortcomings. For example, the majority of registered trials of interventions specified more than one primary outcome; a clinical trial should have one primary outcome, or a combination of co-primary outcomes, but not multiple primary outcomes because primary outcomes are the basis for a sample size estimation. Primary outcomes and outcome measures were very different. Outcomes used in these trials should be used for informing the development of a core outcome set (COS) for COVID-19. It is possible that trialists used multiple primary outcomes that were treated as exploratory due to the early phase of the pandemic.

Various initiatives were already set up to start defining a COS for COVID-19. At least one article about COS-COVID has already been published [15], and multiple initiatives for developing COS for COVID-19 were registered on the web site of the COMET (Core Outcome Measures in Effectiveness Trials) initiative [16].

Many trials mentioned “standard therapy” or “conventional therapy”, and it would be interesting to further investigate what is considered a standard or conventional therapy for a completely new disease with no approved interventions by regulatory agencies. Furthermore, more than 10% of analyzed registered intervention trials were testing hydroxychloroquine and chloroquine, therapies that have been suggested as effective for COVID-19, and that have raised controversies [17].

Accumulation of evidence on COVID-19 is not without challenges. There are particular methodological challenges related to analyzing COVID-19 data during the pandemic [18]. A major challenge is also timely evidence synthesis of the rapidly accumulating data and methodological sacrifices that are being made along the way. Multiple evidence synthesis organizations are now offering evidence collections, investing duplicate effort into similar activities [19]. Overview of systematic reviews published until March 24 indicated that the majority of systematic reviews on COVID-19 available by that date were of critically low methodological quality [20]. Hopefully, research collaborations will be set up to reduce the multiplication of effort in terms of synthesizing and appraising COVID-19 evidence [19].

Early initiatives are evolving and improving along the way. We used WHO collection of evidence on COVID-19, and among the excluded studies there were 4 that were not published in scholarly journals; instead, they were published on a preprint server chemRxiv. Similarly, we have used classification of EPPI-Centre for categorizing analyzed articles into thematic areas; along the way we noticed that the number of articles in their collection had decreased, indicating that they are likely better in curating their content in the living map of evidence [6].

In future studies, it would be worthwhile to continue exploring the growth and characteristics of further studies regarding COVID-19; to analyze how many of the preprint articles will be published in peer-reviewed journals, and how many registered trials will be completed. The resolution of the COVID-19 pandemic is difficult to predict, and this may hinder plans for clinical trials. For countries that may be very successful in their lockdown and quarantine efforts, reduction of the number of infected and diseased patients may prevent the completion of registered clinical trials. Thus, it would be interesting to monitor how many of the registered trials will be terminated prematurely, or will not even begin.

However, in comparison to the past coronavirus epidemics (SARS-CoV and MERS-CoV), the scientific community appears to be much more involved. We were unable to find bibliometric studies comparable to ours about the volume of research considering SARS and MERS, but the simple PubMed search reveals that researchers were much less productive even in the first year after SARS-CoV and MERS-CoV first emerged. Namely, the number of articles from November 1, 2002, to November 1, 2003, and from April 1, 2012, to April 1, 2013, was 611 and 561, respectively.

A limitation of our study is a different search date for the three sources of information we analyzed. However, these sources have major differences in the export functionalities and amount/type of data they provide, and that need to be screened or analyzed. Our analysis of articles published in journal articles took longer time compared to the analysis of preprint articles and registered trials because we needed to conduct screening and analysis about whether those articles contained original data, a quarter of those articles were published in Chinese, and many of those articles were difficult to retrieve from Chinese journals. We are aware that with the ongoing COVID-19 pandemic, research output is fast increasing, but we aimed to analyze early research output, published between 3 and 4 months from the emergence of the new disease.

Furthermore, we did not analyse whether perhaps multiple publications referred to the same dataset. Also, for the translation of non-English articles, we used Google Translate, as it has been shown in 2019 that this tool can be trusted for data extraction in evidence synthesis [7]. One Persian article was additionally clarified through consultation with a native speaker; other languages that are not English were easily translated using Google Translate.

## Conclusion

Early articles on COVID-19 were predominantly retrospective case reports and modelling studies. Many clinical trials about COVID-19 were registered, but it remains to be seen whether they will be completed due to unpredictable development of the pandemic and changes in the number of infected individuals. Diversity of outcomes used in intervention trial protocols indicates the urgent need for defining a core outcome set for COVID-19 research. Chinese scholars had a head start in reporting about the new disease, but publishing articles in Chinese may limit their global reach. Mapping publications with original data can help finding gaps that will help us respond better to the new public health emergency.

## Data Availability

Raw data collected and analyzed within this study are publicly available on Open Science Framework (https://osf.io/dzvxc/).

## Abbreviations

COMET: Core outcome measures in effectiveness trials
JIF: Journal impact factor
OSF: Open science framework
SARS-CoV-2: Severe acute respiratory syndrome coronavirus 2
WHO: World health organization
WHO ICTRP: World health organization international clinical trials registry platform
COVID-19: Coronavirus disease 2019
